# Theaflavin binds to a druggable pocket of TMEM16A channel and inhibits lung adenocarcinoma cell viability

**DOI:** 10.1016/j.jbc.2021.101016

**Published:** 2021-07-28

**Authors:** Sai Shi, Biao Ma, Fude Sun, Chang Qu, Hailong An

**Affiliations:** 1 State Key Laboratory of Reliability and Intelligence of Electrical Equipment, Hebei University of Technology, Tianjin, China; 2 Key Laboratory of Electromagnetic Field and Electrical Apparatus Reliability of Hebei Province, Hebei University of Technology, Tianjin, China; 3 Key Laboratory of Molecular Biophysics, Hebei Province, Institute of Biophysics, School of Science, Hebei University of Technology, Tianjin, China

**Keywords:** ion channel, CaCCs, TMEM16A, theaflavin, inhibitor, A01, CaCC_inh_-A01, CaCCs, calcium-activated chloride channels, DMSO, dimethyl sulfoxide, ESP, electrostatic surface potential, HEK293T, human embryonic kidney 293T cells, LA795, lung adenocarcinoma 795 cells, MD, Molecular dynamics, POPC, 1-Palmitoyl-2-oleoyl-sn-glycero-3-phosphocholine, RMSF, root mean square fluctuations, TF, Theaflavin

## Abstract

As a calcium-activated chloride channel regulated by the intracellular Ca^2+^ concentration and membrane potential, TMEM16A has attracted considerable attention and has been proposed as a novel anticancer drug target. We have previously reported that the pocket above the ion conductance pore could be a nonselective inhibitor-binding pocket. However, whether this pocket is druggable remains unexplored. In this study, we performed virtual screening to target the presumed inhibitor-binding pocket and identified a highly effective TMEM16A inhibitor, theaflavin (TF: a tea polyphenol in black tea). Molecular dynamics simulations revealed that theaflavin adopts a “wedge insertion mode” to block the ion conduction pore and induces pore closure. Moreover, the binding mode showed that the TF pedestal plays an important role in pore blockade, and R515, R535, T539, K603, E623, and E633 were determined to be most likely to interact directly with the pedestal. Mutagenesis experiment results corroborated the mechanism through which TF binds to this pocket. Combined with the quantitative calculation results, our data indicated that the three hydroxyl groups on the pedestal may be the most crucial pharmacophores for TMEM16A inhibition by TF. Finally, antitumor experiments revealed that TF could target TMEM16A to inhibit the proliferation and migration of LA795 cells, indicating the potential therapeutic effect of TF on the growth of lung adenocarcinoma with high TMEM16A expression. The successful application of drug screening strategies based on this binding pocket highlights new directions for discovering superior modulators and contributes to the development of novel therapeutics for lung adenocarcinoma.

Ion channels have been recognized as drug targets for treating numerous diseases, including pain, epilepsy, depression, Alzheimer's disease, and various cancers (1). Therefore, regulating ion channel functions and rectifying the pathological changes in these channels induced by genes or diseases remain crucial therapeutic goals. In this regard, identification of drug-binding sites on ion channels can help elucidate the biophysical properties of channels and affords new opportunities for drug design (2). Currently, high-throughput drugs screening based on binding-site structures as targets has gained momentum for ion channel drug discovery (3).

TMEM16A (also known as anoctamin 1, ANO1) is the molecular basis of calcium-activated chloride channels (CaCCs) (4, 5, 6). TMEM16A belongs to the TMEM16 protein family, which contains ten members in mammals, of which TMEM16A and B can mediate chloride permeation in response to an increase in intracellular calcium concentration, whereas the other members are believed to be lipid scramblase with phospholipid flipping functions (7). Studies have revealed that TMEM16A is involved in regulating oocyte fertilization, transport of matter across cell membranes, and smooth muscle contraction (7, 8, 9). TMEM16A is associated with the development and progression of several diseases and can be potentially targeted for treating asthma, neuropathic pain, gastrointestinal dyskinesia, and secretory diarrhea (2, 10). Furthermore, TMEM16A is reportedly overexpressed in numerous cancers. Accordingly, we previously reported that inhibition of the TMEM16A function suppresses the proliferation and migration of lung adenocarcinoma cells (11, 12). Therefore, TMEM16A has been proposed as an anticancer drug target for recent years (13).

Owing to the pathological roles of TMEM16A in diseases, considerable efforts have been made to identify channel-targeting therapeutic agents, which resulted in the discovery of several TMEM16A channel inhibitors (13). Recently, we revealed the molecular mechanism underlying the inhibition of TMEM16A channel currents by a highly potent TMEM16A inhibitor, CaCC_inh_-A01 (A01) (14), and identified its binding pocket (15). The binding pocket was located in the extracellular vestibule area above pores (Fig. 1*A*). We observed that A01 inhibits TMEM16A channel currents by blocking the ion channel conduction pores. However, the mechanism of inhibition of a substantial number of other TMEM16A channel inhibitors remains unclear, and there is a lack of efficient and safe channel inhibitors (10). A study revealed that more than 50% of the 1221 new small molecules approved by the US Food and Drug Administration between 1981 and 2014 are directly or indirectly derived from natural metabolites (16). Therefore, large-scale drug screening based on natural product databases may be an effective strategy to identify highly potent and safe TMEM16A inhibitors.

**Figure 1:**
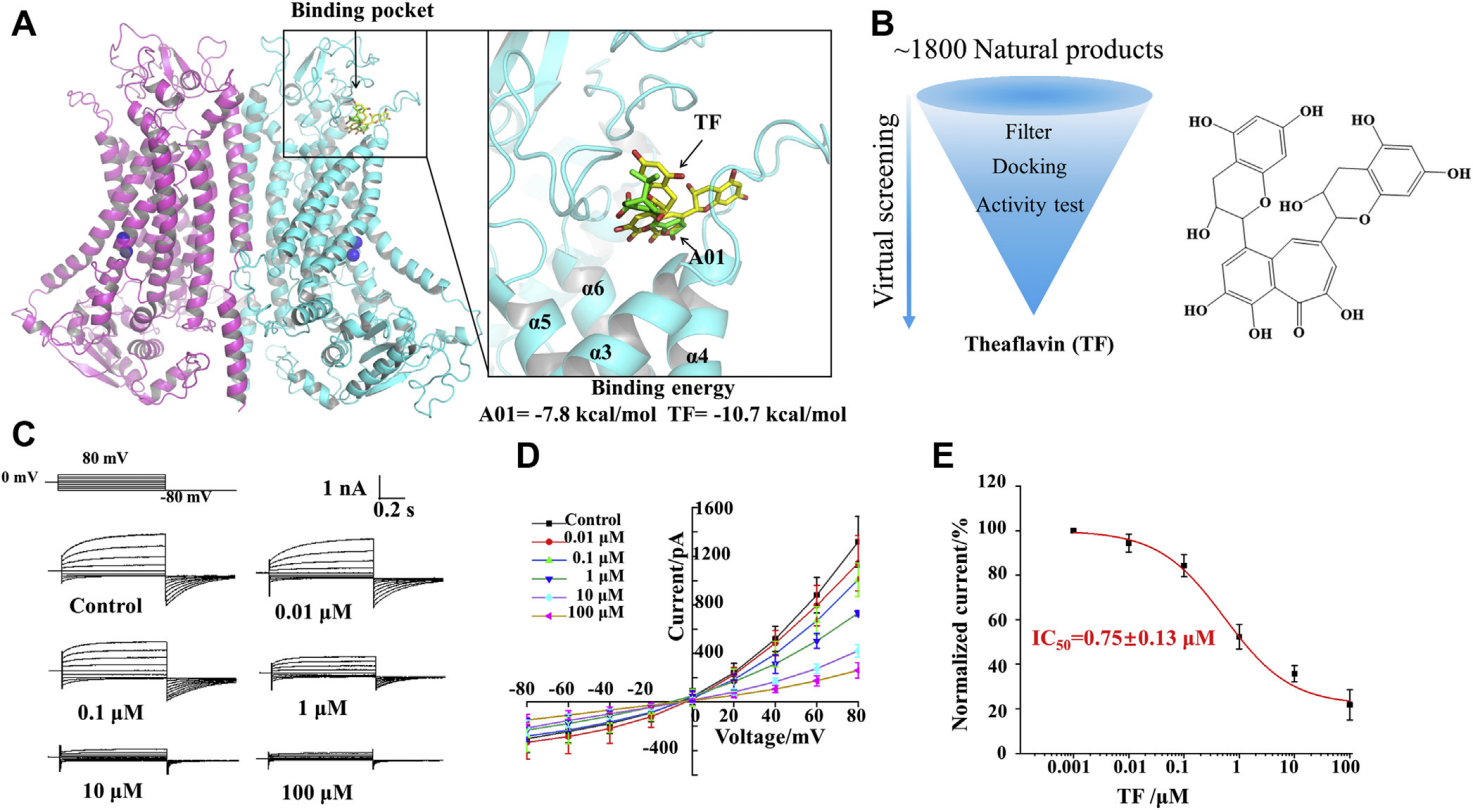
**Discovery of TF as a novel inhibitor of TMEM16A channel.***A*, TMEM16A channel inhibitor-binding pocket. *B*, virtual screening and TF structure. *C*, representative current of TMEM16A inhibited by various concentrations of TF (0, 0.01, 0.1, 1, 10, 100 μM). *D*, I–V curve of inhibition of TMEM16A currents with different concentrations of TF (n = 5). *E*, concentration-inhibition curve of TF inhibition of TMEM16A currents in LA795 cells (n = 5).

To confirm that the binding pocket above the TMEM16A pore is druggable, we screened more than 1800 natural products and identified a potent TMEM16A inhibitor, *i.e.*, theaflavin (TF). Molecular dynamics (MD) simulations detailed the molecular mechanism underlying TMEM16A inhibition *via* TF and determined its binding mode. Moreover, TF effectively inhibited the proliferation and migration of TMEM16A high-expressing lung adenocarcinoma cells, displaying antitumor potential.

## Results

### Screening based on the presumed inhibitor pocket revealed that TF is a novel TMEM16A inhibitor

Previous studies assessing the structure of the TMEM16A channel have indicated that the extracellular vestibule region of the ion conduction pore may be an inhibitor-binding pocket. Small molecules, such as A01, can block pores by directly binding to this pocket (15). To confirm that this binding pocket can be used to discover novel inhibitors, we used Vina to dock a natural product database containing 1827 small molecules. First, we evaluated the affinity of small molecules and proteins using the affinity assessment function of Vina. Then, we selected small molecules to examine their biological activity based on the affinity assessment results. Of note, we observed that TF almost completely inhibited TMEM16A currents at a concentration of 100 μM (Fig. 1, *B* and *C*). Next, a whole-cell patch-clamp was used to evaluate the concentration dependence of the TF inhibitory effect on the TMEM16A current. As shown in Figure 1*C*, the initial recombinant TMEM16A current in human embryonic kidney 293T (HEK293T) cells was activated by 600 nM Ca^2+^ in the pipette solution and then inhibited by different TF concentrations in the bath solution. The current–voltage (I–V) curve of TMEM16A revealed that inhibition of TMEM16A currents by TF did not affect the characteristics of outward rectification and slow activation (Fig. 1*D*). The concentration–response curve was fitted using the Hill function, revealing that the concentration giving half-maximal inhibition (IC_50_) of TF for the inhibition of TMEM16A-mediated currents was 0.75 ± 0.13 μM (Fig. 1*E*). This suggests that the pocket above the pore can be utilized to screen potential channel inhibitors.

### TF not only blocks the pore but also closes the neck region of the pore

**Figure 2:**
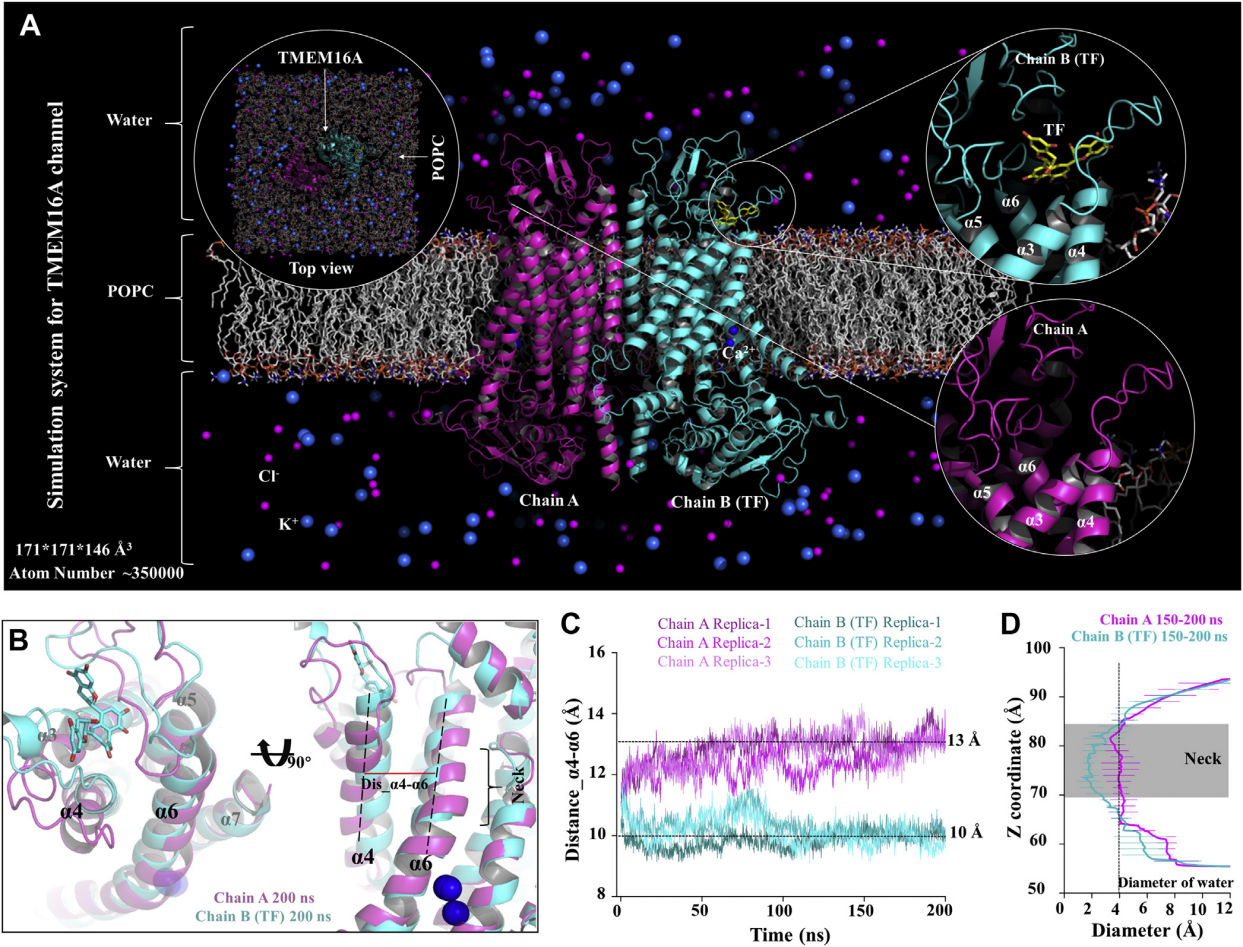
**TF can close the neck of the pore.***A*, molecular dynamics simulation of the TMEM16A system. TMEM16A shown in *cartoon*; POPC is shown in *stick* (*gray*). *B*, the structure overlap of chain A and chain B. *Magenta* for chain A, *cyan* for chain B. *C*, the center of mass distance between α4 and α6. *D*, the pore diameter of the average structure in the 150- to 200-ns stage calculated using HOLE.

To investigate the molecular mechanism of TMEM16A inhibition mediated by TF, we constructed a complex simulation system (including TMEM16A, TF, 1-palmitoyl-2-oleoyl-sn-glycero-3-phosphocholine (POPC), water, and 0.15 mol KCl) (Fig. 2*A*). We selected the pose with the highest affinity (binding energy = −10.3 kcal/mol) between TMEM16A and TF as the initial structure of the dynamic simulation and performed three independent 200-ns MD simulations. It should be noted that TMEM16A is a homodimeric protein, so the system contains two subunits, chain A and chain B, each with its own independent ion conduction pore. TF was docked in chain B, whereas chain A served as a control system without TF. To evaluate the conformational stability of the complex system during MD simulations, we calculated the root mean square deviation (RMSD) of the protein and ligand. As shown in Fig. S1, RMSD values of the protein backbone atoms ranged between 2.86 and 2.99 Å, relative to the initial structure. The RMSD values of the TF heavy atoms ranged between 1.20 and 1.63 Å. This indicated that TF was always bound above the pores of chain B and blocked the ion conduction pores. Several studies have shown that the opening of TMEM16 family proteins was accompanied by the separation of α4 and α6 (17, 18, 19). To analyze the effect of bound TF on the dynamic behavior of the protein, we compared the conformations of chain A and chain B and calculated the distance between α4 and α6. The data showed that the center of mass distance between α4 and α6 for channel containing TF decreased from 11 Å to about 10 Å; the center of mass distance between α4 and α6 for channel without TF increased from 11 Å to about 13 Å (Fig. 2, *B* and *C*). In addition, we calculated the pore diameter of the average structure in the 150- to 200-ns stage of all systems. The data showed that the diameter of the neck region of the pore containing TF was about 1.5 Å, which indicates that the pore was in the closed state. The diameter of the neck region of the pore without TF is about 4 Å, which indicates that the pore is in the preopen state of permeable water molecules (Fig. 2*D* and Fig. S2) and no chloride ion permeation behavior was observed during the simulation. These results suggested that the molecular mechanism of TMEM16A inhibition as mediated by TF is similar to that mediated by A01, inducing pore blockade and leading to pore closure.

### TF pedestal played a key role in blocking pores

**Figure 3:**
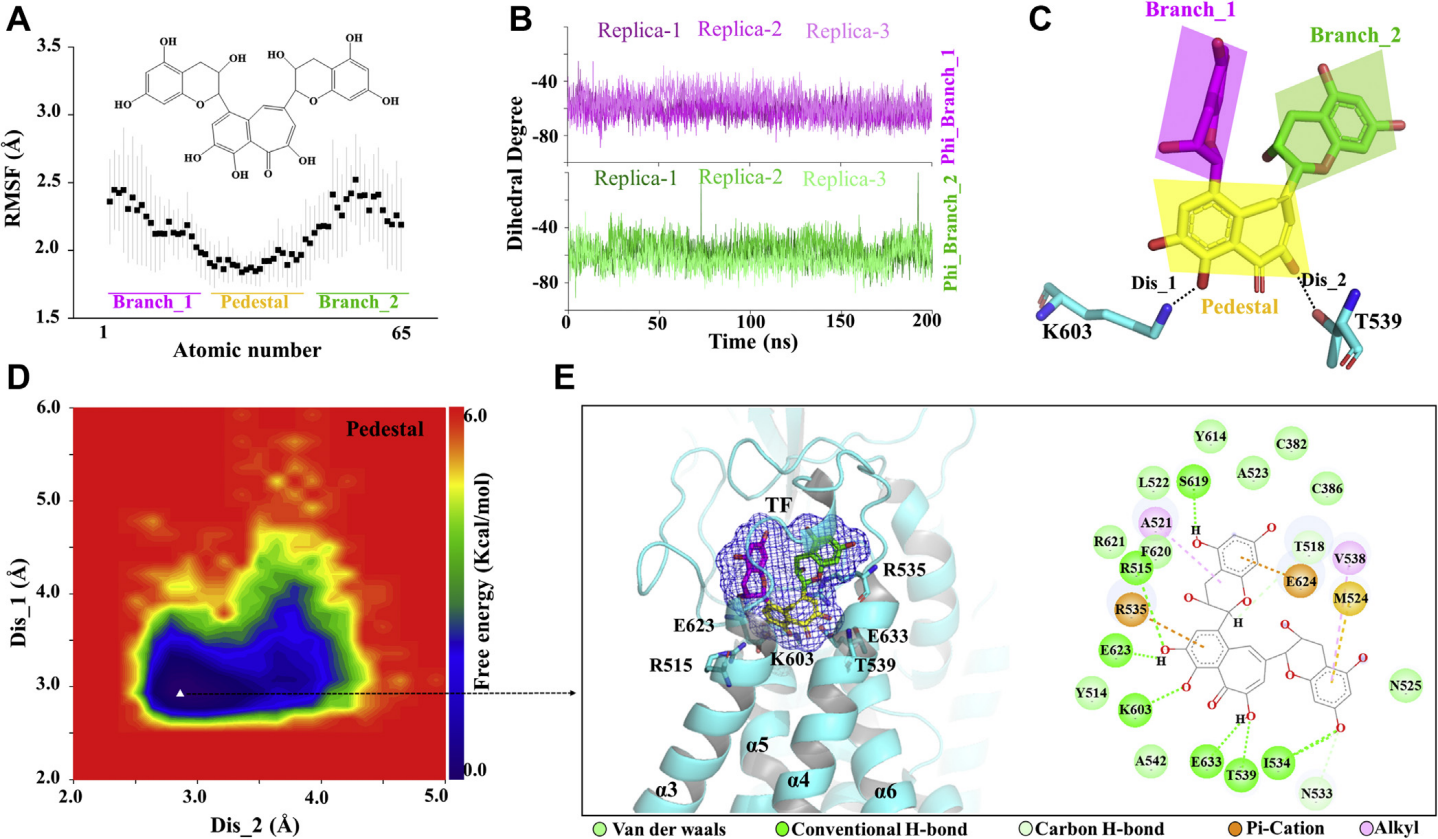
**Binding mode of TF and TMEM16A.***A*, the root means square fluctuation (RMSF) of all atoms of TF. *B*, the dihedral angle diagram of the branches and changes with time. *C*, schematic diagram of the structure of TF. *D*, free energy landscape of the TF binding mode. *E*, representations of the binding mode of the TF and TMEM16A in their lowest binding energy conformation.

Next, we analyzed the dynamic behavior of TF to determine the interaction mode between TF and TMEM16A. First, we calculated the root mean square fluctuations (RMSFs) of all TF atoms to determine their mobility. As shown in Figure 3, *A* and *C*, the RMSF value of the TF pedestal was approximately 1.75 Å, and the RMSF value of the two branches was nearly 2.4 Å. Then, we calculated the dihedral angles of the two branches relative to the pedestal, revealing that the dihedral angles fluctuated between −40°and −80° (Fig. 3, *B* and *C*). This indicated that the TF pedestal was strongly bound in the binding pocket, with the two branches showing high flexibility. To determine the binding mode of TF to TMEM16A, we mapped the free energy landscape of the TF conformation. First, we calculated the distances between the two phenolic hydroxyl oxygen atoms on the pedestal and the side chain oxygen/nitrogen atom of the T539/K603, respectively. We used these data as reaction coordinates to determine the lowest energy conformation of TF (Fig. 3*D*). Data were obtained from three trajectories. We checked the most stable bonding method and observed that the TF pedestal was inserted into the pore entrance like the head of the “wedge,” whereas the two branches were exposed above the pore entrance; this binding mode was termed “wedge insertion mode” (Fig. 3*E*). The interaction mode showed a total of five types of interactions between TF and the 24 residues in the binding pocket, including van der Waals, H-bond interactions, carbon H-bonds, Pi-cations, and alkyl interactions. Among them, H-bond interactions were formed between the hydroxyl group on the pedestal and R515, T539, K603, E623, and E633. Pi-cation interactions were formed between the benzene ring on the pedestal and R535 (Fig. 3*E*). R535 and T539 are located at α4, and it is possible that TF prevents the movement of α4 away from α6 by binding directly to them. The metastable binding mode is similar to the most stable binding mode. The hydroxyl group of the pedestal forms three H-bond interactions with K603/E623/E633 (Fig. S3). Of note, the binding pocket is evolutionarily conserved, especially the several residues bound to the pedestal, R515/K603/E623/E633, showing high conservation (Fig. S4).

### Three hydroxyl groups on the TF pedestal may be critical pharmacophores for TMEM16A inhibition

**Figure 4:**
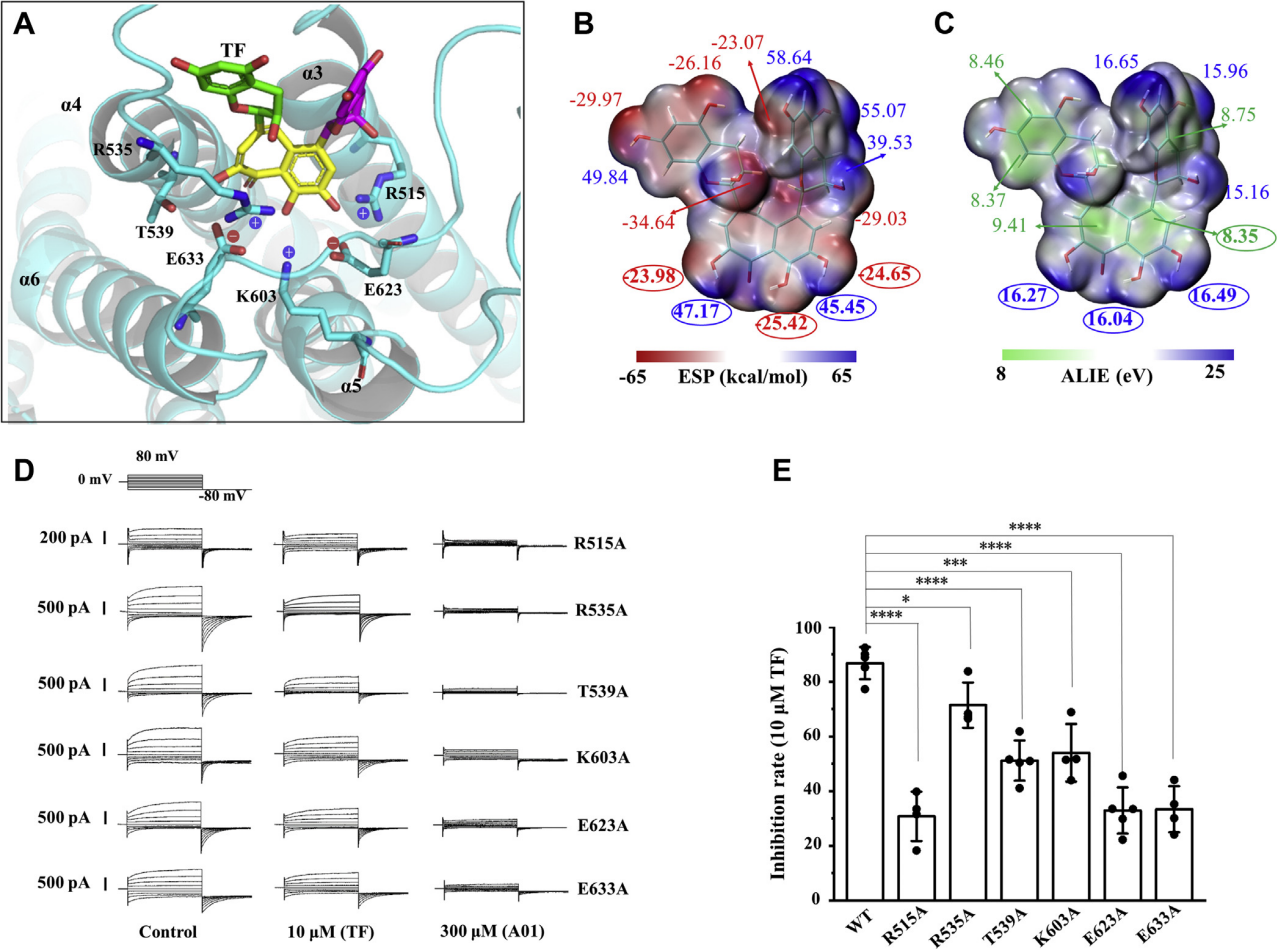
**Binding mode of the TF pedestal and binding pocket.***A*, binding mode of TF to the channel. *B*, ESP-mapped molecular VDW surface of TF. *C*, average local ionization energy (ALIE) of the molecular surface of TF. The colors correspond to the following range of values: *blue* > *white* > *red* (ESP), *blue* > *white* > *green* (ALIE). *D*, representative mutant TMEM16A whole-cell current of HEK293T cells activated by 600 nM Ca^2+^ in the pipette solution and inhibited by 10 μΜ TF and 300 μΜ A01. *E*, inhibition rate of 10 μΜ TF on TMEM16A wildtype and mutant inhibition rate = (I_WT_ − I_10 μΜ TF_)/(I_WT_ − I_300 μΜ A01_). ∗*p* < 0.05, ∗∗∗*p* < 0.001 and ∗∗∗∗*p* < 0.0001 by correlated one-way ANOVA followed by Tukey HSD test (F = 126.637, *p* < 0.0001, R515A *versus* WT; F = 10.611, *p* = 0.014, R535A *versus* WT; F = 71.007, *p* < 0.0001, T539A *versus* WT; F = 35.354, *p* = 0.0005, K603A *versus* WT; F = 136.289, *p* < 0.0001, E623A *versus* WT; F = 125.183, *p* < 0.0001, E633 *versus* WT). n = 4 to 5; Data are means ± SD.

We performed molecular mutagenesis experiments to verify the binding mode. As shown in Figure 4*A*, the pedestal was surrounded by residues R515, R535, T539, K603, E623, and E633, and we sequentially mutated them to alanine to evaluate the inhibitory efficiency of TF on the mutant. First, our results revealed that the TMEM16A mutant could be activated by 600 nM of free calcium ions. Then, we applied 10 μM TF to mutant cells separately activated by calcium ions. The data showed that TF inhibited the mutant currents by 31% (R515A), 71% (R535A), 51% (T539A), 54% (K603A), 33% (E623A), and 33% (E633A), respectively (Fig. 4, *D* and *E*), whereas the wildtype assay demonstrated that 10 μM TF inhibited TMEM16A currents by 87%. R515A, R535A, K603A, E623A, and E633A strongly attenuated TMEM16A inhibition mediated by TF (Fig. 4, *D* and *E*), indicating the essential role of H-bonds between these residues and the hydroxyl group to maintain TMEM16A inhibition mediated by TF. Therefore, the three hydroxyl groups on the pedestal may be important pharmacophores for TMEM16A inhibition by TF. To reveal the physicochemical properties of TF pharmacophores, we calculated the electrostatic surface potential (ESP) and average local ionization energy of the small molecule. As shown in Figure 4, *B* and *C*, hydrogen and oxygen atoms of all phenolic hydroxyl groups of TF are the maximum and minimum value sites of ESP, respectively, and are prone to form hydrogen bonds with negatively and positively charged residues, respectively. Moreover, average local ionization energy values on the seven-membered TF ring and all benzene rings were minimum value sites, indicating high electron activity on these rings. Therefore, they were prone to electron transfer with the receptor, thus forming intermolecular dipole–dipole interactions (Fig. 4, *B* and *C*). The ESP of the three hydroxyl groups on the TF pedestal were complementary to those residues in the binding pocket in terms of electrical charge; this suggested that electrostatic interactions are critical for TMEM16A inhibition by TF (Fig. 4, *A*–*C*).

### TF significantly inhibited the proliferation and migration of LA795 cells

**Figure 5:**
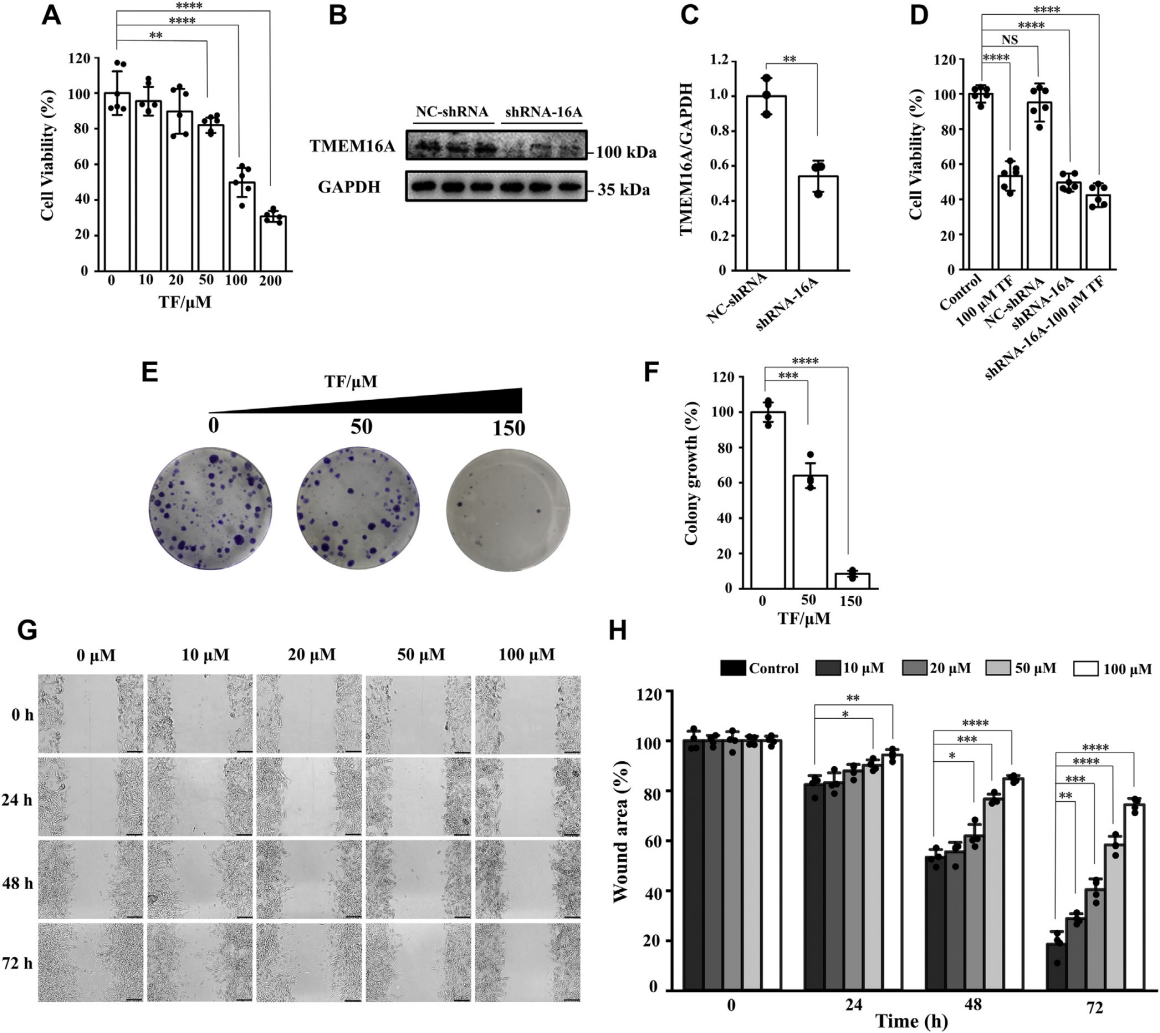
**TF significantly inhibited proliferation and migration of LA795 cell.***A*, inhibitory effect of TF to the proliferation of LA795. ∗∗*p* < 0.01 and ∗∗∗∗*p* < 0.0001 by correlated one-way ANOVA followed by Tukey HSD test (F = 11.90, *p* = 0.006, 50 *versus* 0 μM; F = 71.521, *p* < 0.0001, 100 *versus* 0 μM; F = 185.144, *p* < 0.0001, 200 *versus* 0 μM). n = 6; data are means ± SD. *B*, western blot images of TMEM16A expression in LA795 cells. *C*, the expression of TMEM16A was normalized to the expression level of GAPDH. ∗∗*p* < 0.01 by correlated one-way ANOVA followed by Tukey HSD test (F = 33.885, *p* = 0.004, NC-shRNA *versus* shRNA-16A). n = 3; data are means ± SD. *D*, statistics results of cell viability after transfection with shRNA-16A. ∗∗∗∗*p* < 0.0001 and nonsignificant by correlated one-way ANOVA followed by Tukey HSD test (F = 205.822, *p* < 0.0001, 100 μM TF *versus* control; F = 1.533, *p* = 0.244, NC-shRNA *versus* control; F = 448.740, *p* < 0.0001, shRNA-16A *versus* control; F = 424.188, *p* < 0.0001, shRNA-16A-100 μM TF *versus* control). n = 6; data are means ± SD. *E*, long-term colony formation assay was performed over 2 weeks on lung cancer cell lines with LA795 (*purple*), cultured with the indicated concentrations of TF. *F*, a statistical histogram of (*E*). ∗∗∗*p* < 0.01 and ∗∗∗∗*p* < 0.0001 by correlated one-way ANOVA followed by Tukey HSD test (F = 47.327, *p* = 0.004, 50 μM TF *versus* control; F = 736.609, *p* < 0.0001, 150 μM TF *versus* control). n = 4; data are means ± SD. *G*, migration of LA795 cells in the presence of 0, 10, 20, 50, and 100 μM TF assessed by wound healing assay. Scale bar, 100 μm. *H*, statistical results of wound area in (*D*). ∗*p* < 0.05, ∗∗*p* < 0.01, ∗∗∗*p* < 0.001, and ∗∗∗∗*p* < 0.0001 by correlated one-way ANOVA followed by Tukey HSD test (72 h: F = 14.199, *p* = 0.009, 10 μM TF *versus* control; F = 43.581, *p* = 0.0006, 20 μM TF *versus* control; F = 168.084, *p* < 0.0001, 50 μM TF *versus* control; F = 386.957, *p* < 0.0001, 100 μM TF *versus* control). n = 4; data are means ± SD.

Several studies have shown that TMEM16A overexpression is closely associated with cell proliferation, metastasis, and apoptotic sensitivity in cancer tissues (20, 21, 22); therefore, inhibiting the TMEM16A function may be beneficial in treating related cancers (11, 12). To investigate whether TF has anticancer potency, we selected lung adenocarcinoma 795 (LA795) cells, a lung cancer cell line with high TMEM16A expression (23), and performed the following experiments. MTT assay results revealed that 50 μM TF in the medium significantly inhibited LA795 cell viability. On increasing the TF concentration to 100 and 200 μM, LA795 cell viability decreased to 49.82% and 30.79%, respectively (Fig. 5*A*). To further determine that TF inhibition of TMEM16A can inhibit the activity and proliferation of LA795 cells, we knocked down endogenous TMEM16A expression in LA795 cells using shRNA and performed MTT experiment (Fig. 5, *B* and *C*). Data showed that knockdown of endogenous TMEM16A in LA795 cells resulted in a significant decrease in cell viability (LA795 cell viability decreased to 49.52%), whereas addition of 100 μM TF decreased cell viability by only about 7% (LA795 cell viability decreased to 42.3%) (Fig. 5*D*). Thus, TMEM16A knockdown significantly reduced the biological effect of TF. The above data suggested that TMEM16A is an important target for TF to inhibit LA795. In addition, we performed a colony formation assay using the LA795 cell line to determine whether TF would decrease colony growth. Based on ANOVA, 50 and 150 μM TF could significantly affect colony growth of LA795 cells, with inhibition efficiencies of 36% and 93%, respectively (Fig. 5, *E* and *F*). A wound healing assay was performed to evaluate the effect of TF on the migration of LA795 cells. Different concentrations of TF were added to medium with LA795 cells, and the cells were observed for 72 h. As shown in Figure 5*G*, only 18.41% of the wound area could be observed in the control group after 72 h. However, in the 100 μM TF group, the wound area occupied 74.33% of the total area after 72 h. Figure 5*H* presents the statistical results of the inhibitory effects of different TF concentrations on LA795 cell migration. The above experiments revealed that TF could inhibit the proliferation and migration of LA795 lung cancer cells and is a potential anticancer lead compound.

## Discussion

In recent years, the development of potential modulators as drugs has emerged as a promising new approach for the treatment of various disorders associated with ion channel dysfunction (2). However, the discovery of efficient and safe novel channel modulators remains challenging. In the present study, we confirmed an TMEM16A inhibitor-binding pocket and identified a natural product, TF, that significantly inhibited TMEM16A currents. We also revealed the molecular mechanism through which TF inhibits TMEM16A. Furthermore, our results showed that TF-induced TMEM16A inhibition might contribute to the development of a therapeutic strategy for lung adenocarcinoma. Of note, TF belongs to dietary polyphenolic compounds, which are generally deemed safer than conventional drugs (24), making TF a highly promising antitumor lead compound.

Numerous inhibitors of the TMEM16A channel have been identified, among which A01 demonstrates excellent pharmacokinetics (13). In 2020, we reported the molecular mechanism underling A01-mediated TMEM16A inhibition; we showed that A01 binds to the extracellular vestibule region of the pore, thus blocking the entrance above the pore (15). Therefore, we presumed that the binding pocket above the pore could be used to further discover or design TMEM16A channel inhibitors. The discovery of TF suggested that the binding pocket was druggable. TF is one of the principal constituents of black tea, known to considerably influence the color, fragrance, and characteristic taste of black tea (25). TF has a wide range of biological activities, such as reducing the incidence of cardiovascular disease, as well as antiviral, antibacterial, antiallergic, and antitumor activities (24). TF reportedly possesses an excellent antioxidant capacity, scavenging free radicals and downregulating intracellular reactive oxygen species levels. This induces changes in the expression levels of cell cycle regulatory proteins, resulting in cell cycle arrest and cancer cell death (26, 27). However, the antitumor mechanism of TF may be complex. We found that TF could target TMEM16A channel to inhibit the proliferation and migration ability of LA795 cells that highly express TMEM16A protein. This suggests that TF, as an ion channel blocker, can directly act on cell membrane ion channel to inhibit cancer cell activity. Therefore, based on the results of the present study, we propose that TF could play a key role in TMEM16A-related anticancer effects.

We used MD simulations and molecular mutagenesis to elucidate the molecular mechanism by which TF inhibits TMEM16A. First, electrophysiological experiments and mutagenesis results showed that TF could inhibit TMEM16A channel currents in a concentration-dependent manner and mutations could significantly affect the TF efficacy in inhibiting TMEM16A. Second, modeling the dynamics of TF binding to the TMEM16A channel showed that bound TF blocked the ion conduction pore of TMEM16A and caused α4 and α6 to approach each other, thus resulting in the closure of the pore neck region. Of interest, the binding mode analysis showed that TF adopts a pedestal-down/branch-up binding pose. Accordingly, the pedestal of the molecule is inserted into the pore entrance while the two branches are exposed to the extracellular vestibule; this “wedge insertion mode” leads to a more robust binding of the pedestal to the protein, as verified by the RMSF value of the molecule.

TF is very susceptible to oxidation by prolonged exposure to the environment, which results in an effective concentration of its antiproliferative activity much higher than its IC_50_. Accordingly, to utilize the antitumor action of TF, the bioavailability of TF needs to be improved while overcoming its susceptibility to oxidation (26). We calculated the drug-likeness and bioavailability of TF and observed that the polarity, saturation, and size of TF molecules were beyond the range of theoretical values, resulting in lower gastrointestinal absorption (Fig. S5). Future investigations need to optimize TF considering these three perspectives. Previous studies have shown that the galloyl group of TF is vital for the antioxidant properties of TF (26). We calculated the ESP of the TF molecule using quantum chemical calculations and observed that the galloyl group is the site of the lowest ESP for the whole molecule; moreover, it is highly susceptible to electrophilic reactions. The ESP for the carbonyl oxygen at the pedestal and the neighboring hydroxyl hydrogen atom is complementary; accordingly, intramolecular hydrogen bonds may exist between them. Of importance, the electrostatic features of the TF pedestal were complementary to relevant sites inside the binding pocket, and the alanine mutations in R515, T539, K603, E623, and E633 significantly attenuated the ability of TF to inhibit TMEM16A currents, which not only validated the TF-binding site but also revealed that the phenolic hydroxyl group of the TF pedestal might be a key pharmacophore responsible for inhibition of TMEM16A channel. However, the phenolic hydroxyl group is also a chemical group that is easily oxidized. These findings revealed which groups need to be protected in TF improvement and optimization.

The discovery of TF validates our earlier postulation that the pocket above the TMEM16A channel pore is druggable. This pocket offers a unique opportunity for the future discovery and design of TMEM16A inhibitors. First, it will significantly increase the probability of identifying novel inhibitor drugs. Second, the discovery of TF and the molecular mechanism underlying its TMEM16A channel inhibition provide critical information for further modulating TF. Finally, the antitumor potential of TF may facilitate intensive research on tea, which could be useful for antitumor drug discovery and adjuvant cancer treatment.

## Experimental procedures

### Virtual screening

The crystal structure of TMEM16A (PDB ID: 5OYB) (28) was used to model the receptor structure, and a few missing short loops were complemented using the SWISS-MODEL server (29, 30, 31, 32, 33). Virtual screening was performed using Vina, a docking program that calculates the binding affinity for receptors and ligands (34). We manually collected information from a database of 1827 natural products from the compound company to ensure that compounds could be obtained. Considering the binding pocket size, we extracted compounds with a molecular weight of 200 to 600 Da for the next step of docking screening. Autodock tools (35) were used to prepare the PDBQT files of TMEM16A and compounds. Polar hydrogens were added, and Gasteiger partial charges were assigned to the receptor. The receptor was programmed to remain rigid, whereas the ligand was flexible. The grid center was determined according to the center of the upper binding pocket of the pore, with a searching space size of 24 Å^3^. The global search exhaustiveness value was set to 50. The maximum energy difference between the optimal binding mode and the worst case was set to 5 kcal/mol to ensure diverse docked poses. Natural product monomers purchased from Chengdu DeSiTe Biological Technology Co, Ltd.

### Molecular dynamics simulations

The initial TMEM16A structure was first inserted into model POPC lipid bilayers and then solvated in TIP3P water using the CHARMM-GUI web server (36), during which water molecules are automatically filled into the pores, and the unreasonable water molecules added were manually checked and deleted. Then, 150 mM KCl was added to the solvated system. The final simulation boxes contained approximately 350,000 atoms and dimensions of ∼171 × 171 × 146 Å^3^. All simulations were performed using Amber16 (37). The Amber ff14SB force field, the lipid14 force field, and the Joung/Cheatham ion parameters were used (38). Parametrization of TF was performed using the Antechamber module of Amber16, using the Generalized Amber Force Field to assign atom types and the AM1-BCC method to assign charges.

First, the simulation systems were minimized for 30,000 steps. Next, the temperature was gradually increased from 0 to 300 K in 300 ps under the NVT ensemble with restraint applied to backbone atoms (10 kcal mol^−1^ Å^−2^). An NPT equilibration of 500 ps was then performed, during which the water molecules further fill the pores and protein C_α_ atoms were restrained, with a constraint force of 2 kcal mol^−1^ Å^−2^. Finally, for the simulation system, three independent 200-ns simulations were performed under NPT conditions at 300 K and 1 bar. During all simulations, the backbone of the structured region of the cytosolic domain was harmonically restrained at a force constant of 1.0 kcal mol^−1^ Å^−2^ to minimize the effects of missing loop residues on the cytosolic domain.

During the production process, the system used a Langevin thermostat and a Monte Carlo constant pressure device; the system showed an anisotropic pressure coupling with a nonviscous cutoff of 10 Å. Long-range electrostatic interactions were described by the Particle Mesh Ewald algorithm with a cutoff of 12 Å. The van der Waals interactions were cut off at 12 Å. The lengths of hydrogen-containing covalent bonds were constrained using SHAKE, and the MD time step was set to 2 fs. The snapshots were extracted every 100 ps for all equilibrium MD trajectories to calculate statistical distributions. The CPPTRAJ module (37) of the Amber program was used to analyze the generated trajectories. The pore radius was calculated using the HOLE program (39). ConSurf-DB was used to perform an evolutionary conservation assessment of the proteins (40).

### Quantum chemical calculation

Density functional theory was used to perform necessary theoretical calculations. Gaussian 03 (41) was used to calculate the molecular wave function information. Wave function data used in all analyses were generated using the B3LYP/6-31G^∗∗^ level algorithm. Molecular surface analysis maps were generated by Multiwfn (42) and VMD 1.9.2 program (43).

### Cell culture and transfection

HEK293T and LA795 cells were cultured in Dulbecco's modified Eagle's medium with 10% fetal bovine serum (Sijiqing), 100 IU/ml penicillin, and 100 μg/ml streptomycin in a humidified incubator at 37 °C with 5% CO_2_. The cells were transiently transfected with cDNA for mouse TMEM16A (Accession Number NM_178642.5) using x-treme GENE HP DNA Transfection Reagent (Roche). Cells were seeded on 12-mm glass coverslips in a 24-multiwell plate. Following transfection, the cells were maintained in Dulbecco's modified Eagle's medium at 37 °C for 24 h before patch recording. The shRNA-16A plasmid was transfected into cells with the above transfection reagent to knockdown the expression of endogenous TMEM16A in LA795 cells. The following shRNA targeting the mouse TMEM16A gene was used: CCTGCTAAACAACATCATT (2399–2418 nt).

### Electrophysiology

Whole-cell patch clamp experiments were performed using HEK293T cells at room temperature (22–25 °C). The membrane voltage (Vm) was clamped in steps of 20 mV from −80 to +80 mV, followed by −80 mV. The pipette solution contained CsCl, 130 mM; EGTA, 10 mM; MgCl_2_·6H_2_O, 1 mM; Hepes, 10 mM; MgATP, 1 mM. The bath solution contained NaCl, 150 mM; CaCl_2_, 1 mM; MgCl_2_·6H_2_O, 1 mM; glucose, 10 mM; mannitol, 10 mM; Hepes, 10 mM (adjusted to pH 7.4 with NaOH). The free Ca^2+^ concentration was calculated with Ca-EGTA calculator V1.2 (https://somapp.ucdmc.ucdavis.edu/pharmacology/bers/maxchelator/CaEGTA-NIST.htm). All recordings were performed using an EPC10 amplifier controlled by Pulse software with a Digi LIH1600 interface (HEKA). The data were low-pass filtered at 2.9 kHz and sampled at 10 kHz.

### Site-directed mutagenesis

Agilent Primer Design website (https://www.agilent.com/store/primerDesignProgram.jsp) was used to design site-directed mutagenesis primers. Primer synthesis was performed by Sangon Biotech. Mutants were constructed using a QuickChange II site-directed mutagenesis kit (Agilent Technologies) and confirmed by DNA sequencing (Sangon Biotech). The primer design sequence is provided in the Supporting information (Table S1).

### MTT assay

LA795 cells were seeded into each well of a 96-well plate and cultured for 24 h. Cells were then cultured with dimethyl sulfoxide (DMSO) (control) or TF (0–200 μM) for 48 h before performing the MTT (3-(4,5-dimethylthiazol-2-yl)-2,5-diphenyltetrazolium bromide) assay. Cells were incubated with 5 mg/ml MTT (20 μl) solution for 4 h, and the supernatant was discarded. DMSO (150 μl) was added to each well of the 96-well plate and placed on a shaker at 30 rpm for 10 min to fully dissolve the crystals. The sample absorbance was measured at 490 nm using a SpectraMAX i3 spectrophotometer (Molecular Devices). The percentage of cell viability was calculated by dividing the absorbance of the TF-treated group with that of the control group.

### Colony formation assay

LA795 cells were seeded in six-well plates and cultured for 24 h. Colonies were stained with crystal violet after 2 weeks of growth, and the area was measured. The total colony formation area was calculated using the ImageJ software (US National Institutes of Health).

### Wound healing assay

In brief, LA795 cells were grown to achieve 90% confluency in 24-well plates, scraped with a sterile 200-μl tip, and washed twice with phosphate-buffered saline. Then, the cells were incubated with each medium containing 1% fetal bovine serum and treated with THEAFLAVIN at 0, 10, 20, 50, or 100 μM or DMSO. Finally, cells were photographed at 0, 24, 48, or 72 h under an inverted microscope at ×100 magnification. The wound healing area was calculated using ImageJ software, and the relative scratch area was determined as the ratio of the average area in TF-treated cells to that in the control cells.

### Western blot

Different groups of LA795 cells were washed three times in ice-cold phosphate buffer solution and lysed in RIPA buffer with 1X Halt phosphatase and protease inhibitor cocktails (Solarbio). The proteins were separated on 10% sodium dodecyl sulfate–polyacrylamide gels and electroblotted onto a nitrocellulose membrane in 25 mM Tris and 190 mM glycine at 100 V for 2 h at 4 °C. Blots were incubated in 1:1000 dilutions of the corresponding monoclonal antibodies against TMEM16A, GAPDH, for 12 h at 4 °C. The membranes were then probed with the immunoreactivity by adding secondary antibody diluted to 1:5000 detecting it with chemiluminescent HRP detection kit.

### Data analysis

Graphical presentation and data analysis were performed using Origin 8.0. The data are presented as mean ± standard deviation (SD), and the number of replicates is given in figure legends. Statistical significance of the differences between group means was evaluated by one-way analysis of variance (ANOVA) using Tukey's honestly significant difference (HSD) test as a post hoc test; *p* values ≤0.05 were considered statistically significant. Discovery Studio visualizer was used to analyze noncovalent interactions between TF and its binding pocket. Visualization and analysis of model features were performed by VMD (43) and Open-Source Pymol (https://pymol.org).

## Data availability

All data have been included within the article.

## Supporting information

This article contains supporting information.

## Conflict of interest

The authors declare that they have no conflicts of interest with the contents of this article.
