# Supplementary material for: Theaflavin binds to a druggable pocket of TMEM16A channel and inhibits lung adenocarcinoma cell viability
Source: J Biol Chem. 2021 Jul 28;297(3):101016. doi: 10.1016/j.jbc.2021.101016 (PMC8368026; doi:10.1016/j.jbc.2021.101016)
Supplement: Supplemental Figures S1–S5 and Table S1 [file mmc1.pdf]

Supporting Information

Theaflavin binds to a druggable pocket of TMEM16A channel and inhibits lung adenocarcinoma cell Viability

Sai Shi[a,b,c,1], Biao Ma[a,b,c,1], Fude Sun[c], Chang Qu[a,b,c], Hailong An[a,b,c,*]

[a] State Key Laboratory of Reliability and Intelligence of Electrical Equipment, Hebei University of Technology, Tianjin 300401, China

[b] Key Laboratory of Electromagnetic Field and Electrical Apparatus Reliability of Hebei Province, Hebei University of Technology, Tianjin 300401, China

[c] Key Laboratory of Molecular Biophysics, Hebei Province, Institute of Biophysics, School of Science, Hebei University of Technology, Tianjin, 300401, China;

[1] These authors contributed equally to this work.

[*] Corresponding Author: Professor H. An, e-mail: hailong_an@163.com

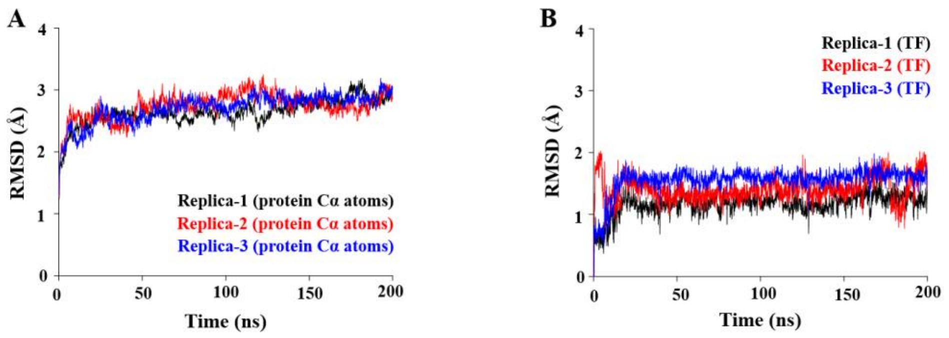

Figure S1. Dynamics behavior of the system. (A) The root means square deviation (RMSD) of the protein. **(B)** The RMSD of the TF heavy atoms.

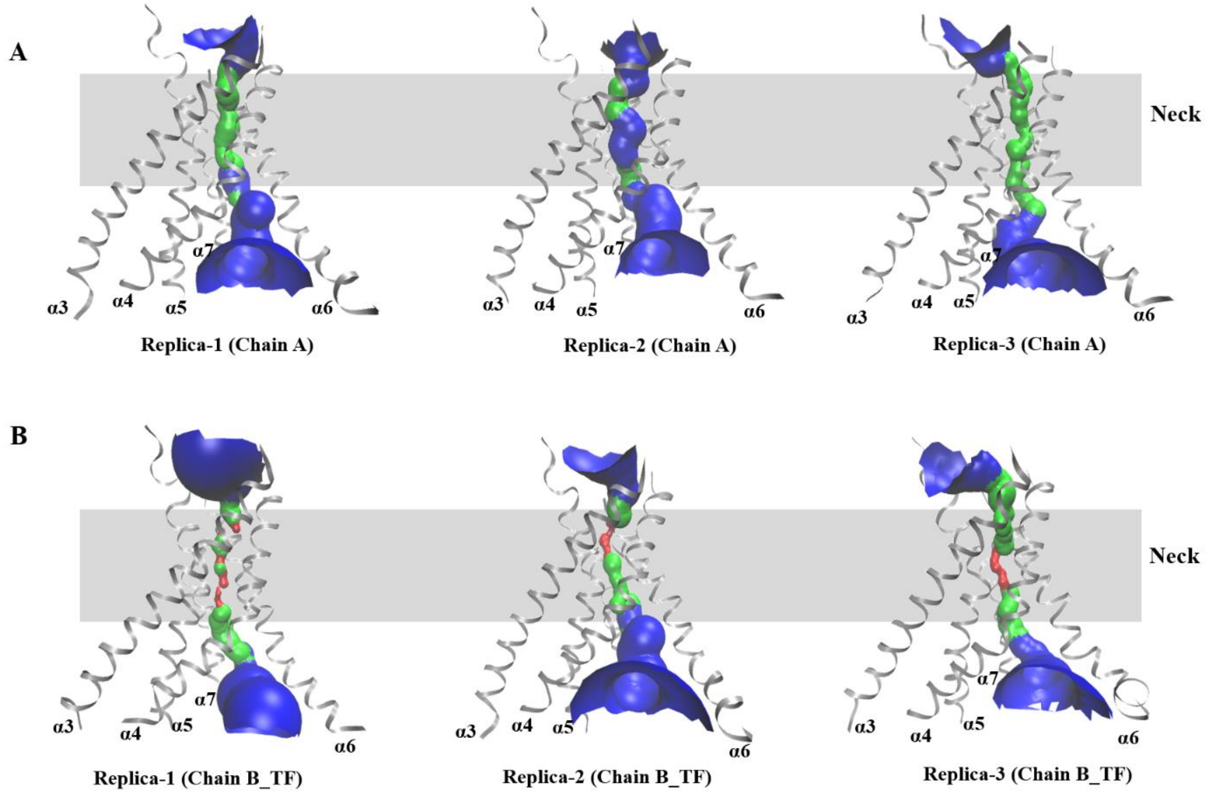

Figure S2. The pore diameter of the average structure in the 150-200 ns stage of all systems. (A) and **(B)** represent the pore maps of chain A and chain B, respectively. The pore maps generated using HOLE is shown as a red (radius < size of water), green (radius ≈ size of water) and blue (radius > size of water).

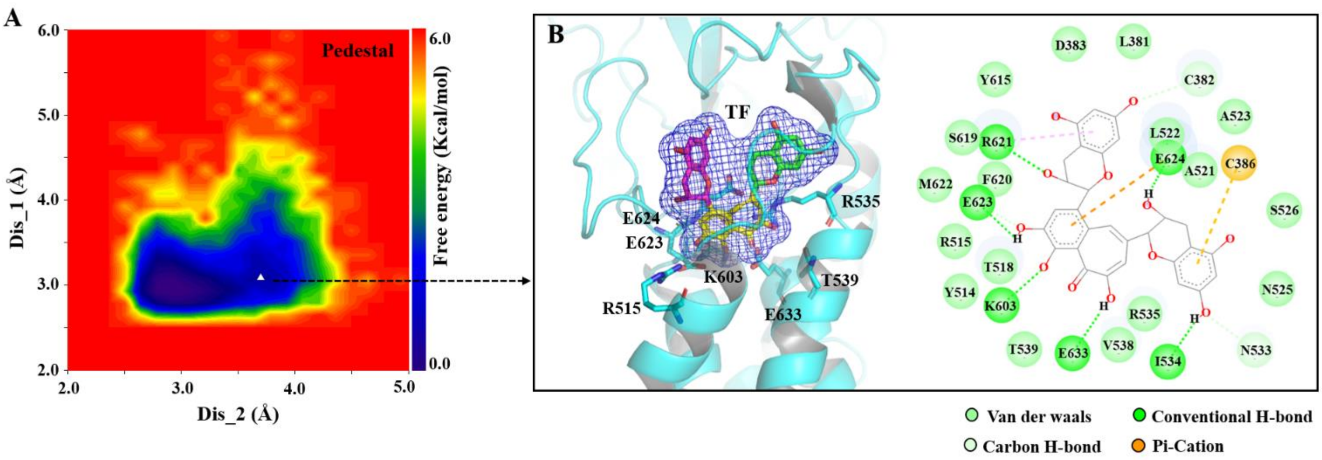

Figure S3. The metastable binding mode of TF and TMEM16A channel. (A) Free energy landscape of the TF binding mode. **(B)** Representations of the metastable binding mode of the TF and TMEM16A.

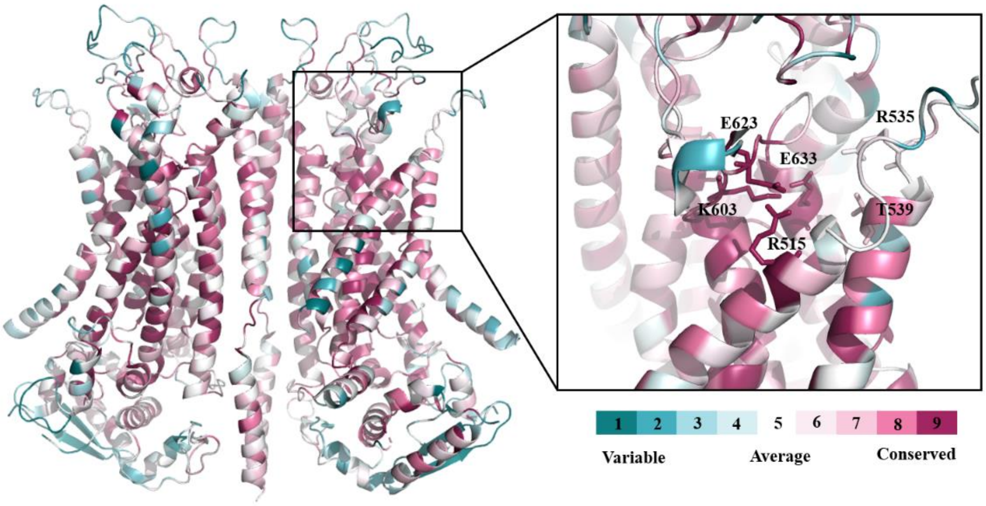

Figure S4. Conservation of TMEM16A channel. Cryo-EM structure of TMEM16A channel (PDB ID: 5oyb), colored by conservation grade and shown in Cartoon representation, together with the key residues, colored by conservation grade and shown as sticks.

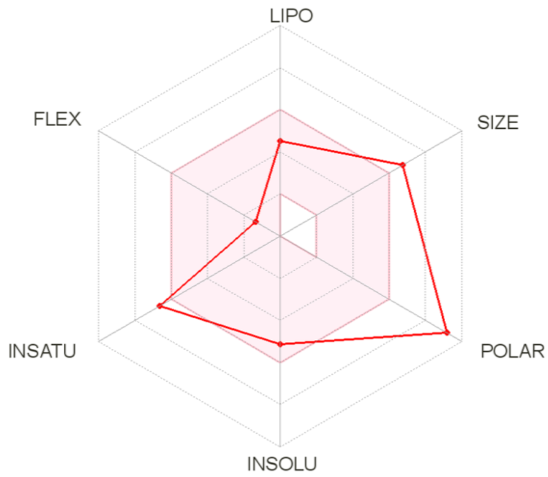

Physicochemical Properties:

Molecular weight	564.49 g/mol
Num. rotatable bonds	2
Num. H-bond acceptors	12
Num. H-bond donors	9
Log Po/w	1.31

Pharmacokinetics:

GI absorption	Low
BBB permeant	No

Druglikeness:

Lipinski	No; 3 violations: MW>500, NorO>10, NHorOH>5
Bioavailability Score	0.17

Figure S5. Drug-likeness of TF. Bioavailability radar considered six physical and chemical properties: lipophilicity, size, polarity, solubility, flexibility and saturation. The physicochemical range on each axis was defined by different descriptors and is depicted as a pink area in which the radar plot of the molecule has to fall entirely into the pink area to evaluate its drug-likeness.

Table S1. The design sequence of the primer.

Primer Name	Sequence (5' to 3')	Number of Nucleobase
**R515A**	cagctgtggagattgcatagatgataactccgaggacgatt	41
**R515Aa**	aatcgtcctcggagttatcatctatgcaatctccacagctg	41
**R535A**	gccgtgactgtaaccgcgatgttggaccgcac	32
**R535Aa**	gtgcggtccaacatcgcggttacagtcacggc	32
**T539A**	cagcggtggccgcgactgtaacccg	25
**T539Aa**	cgggttacagtcgcggccaccgctg	25
**K603A**	gaccaacaaaccggcctgcgaagaaggcgacatagaagatg	41
**K603Aa**	catcttctatgtcgccttcttcgcaggccggtttgttggtc	41
**E623A**	cggggcacactccgccatccggaaaga	27
**E623Aa**	tctttccggatggcggagtgtgccccg	27
**E633A**	gctggatacagagcgccatgaggcagccg	29
**E633Aa**	cggctgcctcatggcgctctgtatccagc	29
